# Interventions and Operations after Bariatric Surgery in a Health Plan Research Network Cohort from the PCORnet, the National Patient-Centered Clinical Research Network

**DOI:** 10.1007/s11695-021-05417-7

**Published:** 2021-04-20

**Authors:** Qinli Ma, Sonali Shambhu, David E. Arterburn, Kathleen M. McTigue, Kevin Haynes

**Affiliations:** 1 Translational Research for Affordability and Quality, HealthCore, Inc., 123 Justison Street, Suite 200, Wilmington, DE 19801-5134 USA; 2 Kaiser Permanente Washington Health Research Institute, Seattle, WA USA; 3 Department of Epidemiology, University of Pittsburgh, Pittsburgh, PA USA

**Keywords:** Bariatric surgery, Roux-en-Y gastric bypass procedure (RYGB), Adjustable gastric banding (AGB), Sleeve gastrectomy (SG), Adverse events

## Abstract

**Purpose:**

Obesity is a highly prevalent condition with severe clinical burden. Bariatric procedures are an important and expanding treatment option. This study compared short-(30-day composite adverse events) and long-term (intervention/operation, endoscopy, hospitalization, and mortality up to 5 years) safety outcomes associated with three bariatric surgical procedures.

**Materials and Methods:**

This observational cohort study replicated an electronic health record study comparing short- and long-term problems associated with three bariatric surgical procedures between January 1, 2006, and September 30, 2015, within a Health Plan Research Network.

**Results:**

Of 95,251 adults, 34,240 (36%) underwent adjustable gastric banding (AGB), 36,206 (38%) Roux-en-Y gastric bypass (RYGB), and 24,805 (26%) sleeve gastrectomy (SG). Median (interquartile range) years of follow-up was 3.3 (1.4–5.0) (AGB), 2.5 (1.0–4.6) (RYGB), and 1.1 (0.5–2.1) (SG). Overall mean (SD) age was 44.2 (11.4) years. The cohort was predominantly female (76%). Thirty-day composite adverse events occurred more frequently following RYGB (3.8%) than AGB (3.1%) and SG (2.8%). Operation/intervention was less likely in SG than in RYGB (adjusted hazard ratio (AHR), 0.87; 95%CI, 0.80–0.96; *P*=0.003), and more likely in AGB than in RYGB (AHR, 2.10; 95%CI, 2.00–2.21; *P*<0.001). Hospitalization was less likely after ABG and SG than after RYGB: AGB vs. RYGB, AHR=0.73; 95%CI, 0.71–0.76; *P<*0.001; SG vs. RYGB, AHR=0.79; 95%CI, 0.76–0.83; *P*<0.001. Mortality was most likely for RYGB (SG vs. RYGB: AHR, 0.76; 95%CI, 0.64–0.92; *P*=0.004; AGB vs. RYGB: AHR, 0.49; 95%CI, 0.43–0.56; *P*=0.001).

**Conclusions:**

Interventions, operations, and hospitalizations were more often associated with AGB and RYGB than SG while RYGB had the lowest risk for revision.

**Graphical abstract:**

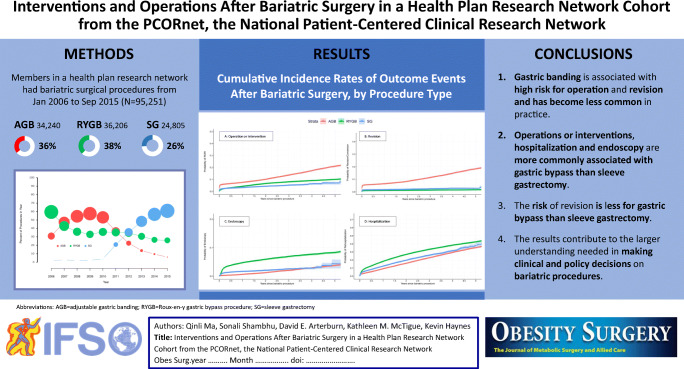

**Supplementary Information:**

The online version contains supplementary material available at 10.1007/s11695-021-05417-7.

## Introduction

An estimated 93.3 million American adults were affected by obesity in 2015–2016; the estimated prevalence rate in adults was 39.8%, and 18.5% in youths, with a substantial portion severe enough (body mass index > 35 kg/m^2^ ) to merit consideration of bariatric surgery [1]. Because of its already high and increasing prevalence rate, obesity is deemed an epidemic by the Centers of Disease Control and Prevention [2]. Obesity is linked to several leading causes of preventable death including cardiovascular disease, type 2 diabetes, and some cancers, exacting a severe clinical burden [3].

As the prevalence of obesity increased, bariatric procedures became an important and rapidly expanding part of the treatment arsenal [4–6], with evidence shown to be superior to medical and lifestyle interventions for weight loss and glycemic control [7–12]. Roux-en-Y gastric bypass (RYGB) was predominant in the early 2000s; however, by the late 2000s, adjustable gastric banding (AGB) procedure had become more widely used. Both RYGB and AGB appear to have waned in popularity following the emergence of sleeve gastrectomy (SG), which is the most commonly used bariatric procedure currently[13, 14].

Information on the long-term comparative outcomes of these common bariatric procedures is accumulating [4, 6, 15, 16]. Pieces of evidence are wanted to address the existing variation in clinical utilization and insurance coverage of the surgical procedures [17, 18]. To help generate new knowledge and insights on their comparative long-term safety and effectiveness, the National Patient-Centered Clinical Research Network (PCORnet), using electronic health record (EHR) data, analyzed outcomes from three of the most commonly performed bariatric procedures: AGB, RYGB, and SG [19, 20], and have recently reported their results [21–24].

The purpose of the present study was to compare the short- and long-term *safety* outcomes across the three common bariatric surgery procedures, AGB, RYGB, and SG, using administrative claims data and the methodology of the PCORnet Bariatric Study (PBS). This study complements other real-world data to compare the safety of different bariatric procedures in the United States.

## Methods

### Study Design and Data Source

This observational cohort study simulated the research plan and employed the sample selection criteria described in the PBS [19–22]. This study first ran the PBS computable phenotype in the HealthCore-Anthem Research Network (HCARN) claims data stored in the PCORnet common data model. The HCARN data is a proprietary and longitudinal claims database containing medical and pharmacy information from enrollees within 14 US-based regional health plans. The computable phenotype retrospectively identified health plan enrollees who received bariatric surgery (see Supplementary Table 1 for codes) between January 1, 2006, and September 30, 2015. The initial bariatric procedure was defined as the index procedure, and the procedure date was defined as the index date. Adopting the PBS population definition, we evaluated adult patients (20–79 years old at the index date) and stratified them by index procedure type [19–22].

### Outcomes of Interest

We used HCARN claims data in the PCORnet common data model to identify adverse events of interest delineated in the PBS [19–22]. Short-term safety included 30-day composite outcome, including venous thromboembolism, percutaneous or operative intervention, not being discharged within 30 days, or death. The long-term safety outcomes were evaluated up to 5 years after the index procedure. The primary long-term adverse event included subsequent operation or intervention encompassing any additional bariatric procedure and abdominal procedures. The secondary long-term adverse events consisted of subsequent endoscopy, revision (a component subcategory of operations), all-cause hospitalization, and all-cause death. Patients were censored once they were no longer enrolled with health plans or death or as of September 30, 2015, whichever was first. This study used a limited, deidentified dataset and was exempted from informed consent requirements by the New England Independent Review Board.

### Statistical Analysis

Multivariable logistic regression analysis was used to estimate adjusted odds ratios (AORs) to compare the three types of bariatric surgeries for the 30-day composite outcome. Cox proportional hazard models were applied to estimate adjusted hazard ratios (AHRs) and cumulative probability of long-term outcomes for the three surgery types. All baseline demographic and clinical characteristics were adjusted in all models. For exploratory purposes, heterogeneity in treatment effects was tested across gender and age (<65, or ≥65 years), and a subgroup analysis was conducted on those aged 20 to 64 years old. All analyses were conducted with R Studio Pro, version 3.6.3 software.

## Results

There were 95,251 adults that met the eligibility criteria for this study, as shown in Supplementary Figure 1. Overall, the mean (SD) age was 44.2 (11.4) years. The cohort was predominantly female (75.8%). Among eligible patients, 34,240 (35.9%) underwent AGB, 36,206 (38.0%) underwent RYGB, and 24,805 (26.0%) underwent SG. The RYGB group was slightly older (mean: 44.9 vs. 43.7 years for SG, and 43.9 years for AGB) (Table 1), with the highest rates of diabetes (42.4%) and hypertension (70.4%). A total of 1072 deaths occurred during the following and 42,574 patients had 5 years’ follow-up and 51,605 either disenrolled from the health plan or reached the end of the study period (09/30/2015) during the follow-up. The median (interquartile range) follow-up was 3.3 (1.4–5.0) years for AGB, 2.5 (1.0–4.6) years for RYGB, and 1.1 (0.5–2.1) years for SG.

**Table 1:** Baseline characteristics

Characteristic	Patients by operation			All patients	Standardized mean difference		
	AGB (*n*=34,240)	RYGB (*n*=36,206)	SG (*n*=24,805)	*n*=95,251	AGB vs. RYGB	AGB vs. SG	SG vs. RYGB
Age, mean (SD), y	43.9 (11.4)	44.9 (11.5)	43.7 (11.2)	44.2 (11.4)	−0.0934	0.0124	−0.1063
Median	44	45	43	44			
Sex, no. (%)					−0.0443	−0.0511	0.0069
Female	26,399 (77.1%)	27,232 (75.2%)	18,583 (74.9%)	72,214 (75.8%)			
Male	7,841 (22.9%)	8,974 (24.8%)	6,222 (25.1%)	23,037 (24.2%)			
Procedure year, no. (%)					0.4564	2.306	1.4788
2006–2009	17,626 (51.5%)	14,574 (40.3%)	267 (1.1%)	32,467 (34.1%)			
2010	6,317 (18.5%)	4,205 (11.6%)	265 (1.1%)	10,787 (11.3%)			
2011	4,809 (14.0%)	4,643 (12.8%)	2,654 (10.7%)	12,106 (12.7%)			
2012	2,596 (7.6%)	4,102 (11.3%)	4,219 (17.0%)	10,917 (11.5%)			
2013	1,526 (4.5%)	3,644 (10.1%)	5,859 (23.6%)	11,029 (11.6%)			
2014	1,037 (3.0%)	3,237 (9.0%)	7,152 (28.8%)	11,426 (12.0%)			
2015	329 (1.0%)	1,801 (5.0%)	4,389 (17.7%)	6,519 (6.8%)			
Comorbidities, no. (%)							
Anxiety	7,639 (22.3%)	9,366 (25.9%)	7,851 (31.7%)	24,856 (26.1%)	−0.0833	−0.2116	0.128
Depression	10,208 (29.8%)	12,198 (33.7%)	8,583 (34.6%)	30,989 (32.5%)	−0.0834	−0.1026	0.0192
Diabetes	10,798 (31.5%)	15,332 (42.4%)	8,380 (33.8%)	34,510 (36.2%)	−0.2254	−0.0479	−0.177
DVT	346 (1.0%)	615 (1.7%)	362 (1.5%)	1,323 (1.4%)	−0.0596	−0.0407	−0.0192
Dyslipidemia	18,518 (54.1%)	21,320 (58.9%)	13,332 (53.8%)	53,170 (55.8%)	−0.097	0.0067	−0.1037
Eating disorder	3,712 (10.8%)	4,304 (11.9%)	3,410 (13.8%)	11,426 (12.0%)	−0.033	−0.0886	0.0557
GERD	18,557 (54.2%)	22,255(61.5%)	16,181 (65.2%)	56,993 (59.8%)	−0.1476	−0.2264	0.0782
Hypertension	21,802 (63.7%)	25,484 (70.4%)	16,225 (65.4%)	63,511 (66.7%)	−0.1431	−0.0363	−0.1067
Kidney disease	984 (2.9%)	2,105 (5.8%)	1,114 (4.5%)	4,203 (4.4%)	−0.1446	−0.0859	−0.0599
NAFLD	5,381 (15.7%)	8,901 (24.6%)	5,605 (22.6%)	19,887 (20.9%)	−0.2225	−0.1755	−0.0468
Osteoarthritis	1,663 (4.9%)	1,916 (5.3%)	1,401 (5.7%)	4,980 (5.2%)	−0.0198	−0.0355	0.0157
PE	319 (0.9%)	525 (1.5%)	332 (1.3%)	1,176 (1.2%)	−0.0478	−0.1582	−0.0095
Sleep apnea	15,683 (45.8%)	19,379 (53.5%)	13,317 (53.7%)	48,379 (50.8%)	−0.1549	−0.1026	0.0033

AGB=adjustable gastric banding; DVT=deep vein thrombosis; GERD=gastroesophageal reflux disease; NAFLD=nonalcoholic fatty liver disease; PE=pulmonary embolism; RYGB=Roux-en-y gastric bypass; SD=standard deviation; SG=sleeve gastrectomy; y=year

**Fig. 1:**
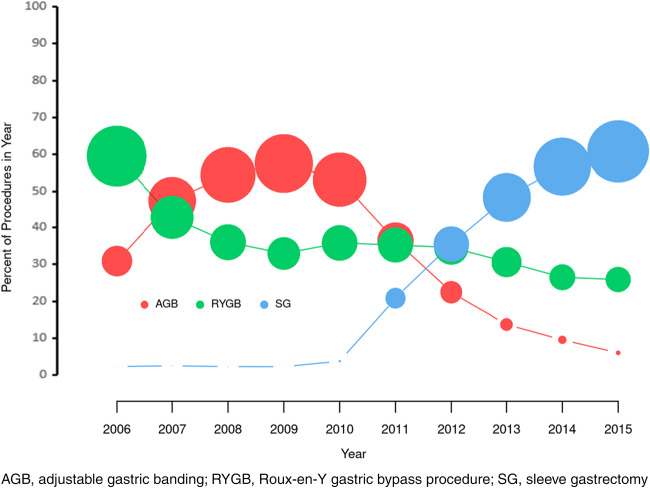
Temporal trends in bariatric procedures from 2006 to 2015. Size of the bubble is proportionate to the number of patients at that time point

Figure 1 shows the temporal trend in bariatric surgeries performed from 2006 to 2015. We observed the peak proportions of patients with AGB in 2009 (63.6%) and a substantial decline to 9.1% in 2014. The proportion undergoing SG increased dramatically from 0.8% in 2006 to 62.6% in 2014. The trend for RYGB was relatively stable.

### 30-Day Composite Outcome

Within 30 days after the index procedure, 3.05% of AGB, 3.80% of RYGB, and 2.78% of SG had the composite outcome (Table 2). Larger proportions required intervention: 2.62% for AGB, 2.14% for RYGB, and 1.71% for SG. The AORs for the composite outcome were significantly lower for AGB relative to RYGB (AOR, 0.81; 95% CI, 0.72–0.92; *P*<0.001) and SG (AOR, 0.80; 95% CI, 0.73–0.87; *P*<0.001). Compared to the RYGB group, the SG group had similar probability (AOR, 0.98; 95% CI, 0.88–1.10; *P*=0.08).

**Table 2:** Major adverse events occurring in the first 30-days after bariatric surgery, by procedure type

30-day adverse events	AGB (*n*=34,240)		RYGB (*n*=36,206)		SG (*n*=24,805)		Total (*n*=95,251)	
	*n*	%	*n*	%	*n*	%	*n*	%
Venous thromboembolism	139	0.41%	307	0.85%	190	0.77%	636	0.67%
Percutaneous or operative intervention	896	2.62%	775	2.14%	425	1.71%	2096	2.20%
Death	6	0.02%	87	0.24%	32	0.13%	125	0.13%
Failure of discharge	6	0.02%	243	0.67%	60	0.24%	309	0.32%
Composite adverse event	1045	3.05%	1377	3.80%	689	2.78%	3111	3.27%

*AGB*, adjustable gastric banding; *RYGB*, Roux-en-Y gastric bypass; *SG*, sleeve gastrectomy

30-day composite adverse event is defined as at least one of the following within 30 days of bariatric surgery: venous thromboembolism, percutaneous or operative intervention, failure of discharge, or death

### Primary Long-Term Outcome

**Fig. 2:**
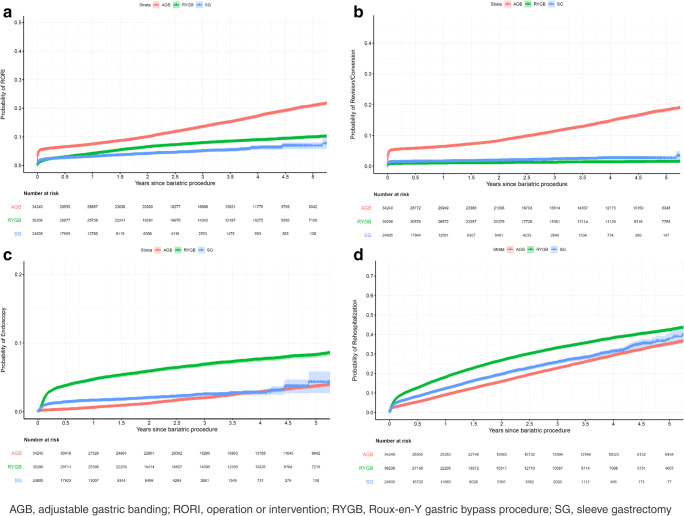
Cumulative incidence rates of outcome events after bariatric surgery, by procedure type

Operation or intervention was more likely following AGB compared to RYGB (AHR, 2.10; 95%CI, 2.00–2.21; *P* <0.001) but less likely for SG than RYGB (AHR, 0.87; 95%CI, 0.80–0.96; *P*=.003) (Table 3). Accordingly, the estimated cumulative probability (95% CI) of operation or intervention was higher for AGB, followed by RYGB and then SG. The probability for AGB was 7.0% (6.7–7.3%) at 1 year, 12.6% (12.1–3.1%) at 3 years, and 18.3% (17.6–19.0%) at 5 years (Table 4, Fig. 2a).

**Table 3:** Adjusted hazard ratios for comparison of different events

Outcome	Procedures	Adjusted hazard ratios	Lower 95% CI	Upper 95% CI	*P* value
Operation or intervention, excluding endoscopy	SG vs. RYGB	0.87	0.80	0.96	0.003
	AGB vs. RYGB	2.10	2.00	2.21	<0.001
	AGB vs. SG	2.40	2.20	2.62	<0.001
Endoscopy	SG vs. RYGB	0.43	0.38	0.48	<0.001
	AGB vs. RYGB	0.36	0.33	0.39	<0.001
	AGB vs. SG	0.84	0.74	0.96	0.01
Revision	SG vs. RYGB	2.88	2.48	3.33	<0.001
	AGB vs. RYGB	11.33	10.24	12.54	<0.001
	AGB vs. SG	3.94	3.50	4.43	<0.001
Hospitalization	SG vs. RYGB	0.79	0.76	0.83	<0.001
	AGB vs. RYGB	0.73	0.71	0.76	<0.001
	AGB vs. SG	0.92	0.88	0.97	0.0011
Mortality	SG vs. RYGB	0.76	0.64	0.92	0.004
	AGB vs. RYGB	0.49	0.43	0.56	0.001
	AGB vs. SG	0.64	0.52	0.79	<0.001

*AGB*, adjustable gastric banding; *CI*, confidence interval; *RYGB*, Roux-en-Y gastric bypass; *SG*, sleeve gastrectomy

**Table 4:** Estimated percentages of patients with outcome event at specified time

	Estimated % (95% CI)		
Outcome	At 1 year	At 3 years	At 5 years
Operation or intervention excluding endoscopy			
RYGB	3.39 (3.23–3.56)	6.23 (5.94–6.51)	9.19 (8.77–9.61)
SG	2.97 (2.75–3.19)	5.47 (5.07–5.86)	8.08 (7.5–8.66)
AGB	7.00 (6.71–7.28)	12.63 (12.15–13.11)	18.33 (17.63–19.03)
Endoscopy			
RYGB	3.65 (3.45–3.84)	6.04 (5.73–6.34)	8.29 (7.86–8.72)
SG	1.57 (1.41–1.74)	2.62 (2.35–2.98)	3.63 (3.25–4.00)
AGB	1.33 (1.22–1.43)	2.22 (2.05–2.38)	3.06 (2.83–3.30)
Revision			
RYGB	0.50 (0.45–0.55)	0.86 (0.77–0.95)	1.41 (1.26–1.56)
SG	1.43 (1.28–1.58)	2.45 (2.19–2.70)	4.01 (3.59–4.43)
AGB	5.50 (5.19–5.82)	9.30 (8.78–9.81)	14.90 (14.07–15.71)
Hospitalization			
RYGB	14.33 (13.99–14.66)	30.04 (29.44–30.63)	42.25 (41.46–43.04)
SG	11.56 (11.14–11.97)	24.70 (23.89–25.49)	35.34 (34.26–36.41)
AGB	10.73 (10.44–11.01)	23.05 (22.51–23.59)	33.16 (32.40–33.90)
Mortality			
RYGB	0.34 (0.30–0.38)	0.64 (0.57–0.71)	0.98 (0.88–1.09)
SG	0.26 (0.21–0.31)	0.49 (0.40–0.57)	0.75 (0.63–0.88)
AGB	0.17 (0.14–0.19)	0.31 (0.27–0.36)	0.48 (0.42–0.55)

*AGB*, adjustable gastric banding; *CI*, confidence interval; *RYGB*, Roux-en-Y gastric bypass; *SG*, sleeve gastrectomy

#### Heterogeneity of Treatment Effects for Primary Outcome and Subgroup Analysis

Heterogeneity of treatment effects was examined across gender (female, male) and age (<65, ≥65 years) (Table 5). There was no evidence of heterogeneity of treatment effects for SG vs. RYGB for gender and age. However, the increased risk of operation or intervention for AGB, compared to RYGB, was lower for males than females (male: AHR, 1.7; 95%CI, 1.6–1.9; and female: 2.2; 95% CI, 2.1–2.3; *P <* 0.001), and lower for age ≥65 than age <65 (age ≥65: 1.2; 95%CI, 1.0–1.6; and age <65: 2.2; 95% CI, 2.0–2.3; *P* < 0.001). Similarly, the increased risk for operation or intervention for AGB, compared to SG, was also lower for males than females and for age ≥65 than age <65 (Table 5). For the subgroup analysis, 91,854 (96.4% of the overall 95,251 patients) aged 20–64 years old, and the results for short- and long-term safety outcomes were similar to the main analysis (Supplementary Table 2).

**Table 5:** Heterogeneity of treatment effects for operation or intervention outcome by gender and age

**Gender**										
**Evidence of heterogeneous treatment effect of procedures on event time given gender**										
	Female				Male					
	Adjusted HR	Lower 95% CI		Upper 95% CI	Adjusted HR	Lower 95% CI		Upper 95% CI	*P* value for HTE	
SG vs. RYGB	0.84	0.76		0.93	0.98	0.84		1.15	0.09	
AGB vs. RYGB	2.21	2.09		2.33	1.74	1.57		1.93	<0.001	
AGB vs. SG	2.63	2.39		2.90	1.77	1.52		2.07	<0.001	
**Estimated percent of patients with event at 1, 3, and 5 years**										
		1 year			3 years			5 years		
		Estimate (%)	Lower 95% CI	Upper 95% CI	Estimate (%)	Lower 95% CI	Upper 95% CI	Estimate (%)	Lower 95% CI	Upper 95% CI
RYGB	Female	3.46	3.28	3.64	6.35	6.03	6.66	9.37	8.89	9.83
	Male	3.23	2.95	3.51	5.93	5.42	6.43	8.76	8.02	9.49
SG	Female	2.91	2.67	3.16	5.35	4.91	5.8	7.92	7.27	8.57
	Male	3.17	2.74	3.6	5.83	5.05	6.59	8.61	7.48	9.73
AGB	Female	7.47	7.15	7.79	13.47	12.93	14	19.5	18.73	20.27
	Male	5.55	5.16	5.94	10.09	9.41	10.77	14.75	13.77	15.72
**Age**										
**Evidence of heterogeneous treatment effect of procedures on event time given age at baseline**										
	Age <65 years				Age ≥65 years					
	Adjusted HR	Lower 95% CI		Upper 95% CI	Adjusted HR	Lower 95% CI		Upper 95% CI	*P* value for HTE	
SG vs. RYGB	0.88	0.80		0.96	0.93	0.61		1.42	0.77	
AGB vs. RYGB	2.15	2.04		2.25	1.24	0.97		1.58	<0.001	
AGB vs. SG	2.45	2.24		2.67	1.33	0.88		2.01	0.004	
**Estimated percent of patients with event at 1, 3, and 5 years**										
		**1 year**			**3 years**			**5 years**		
		Estimate (%)	Lower 95% CI	Upper 95% CI	Estimate (%)	Lower 95% CI	Upper 95% CI	Estimate (%)	Lower 95% CI	Upper 95% CI
RYGB	Age <65	3.37	3.21	3.54	6.19	5.9	6.47	9.13	8.71	9.56
	Age ≥65	3.78	3.08	4.48	6.92	5.67	8.17	10.2	8.37	11.99
SG	Age <65	2.96	2.74	3.19	5.45	5.05	5.84	8.05	7.47	8.64
	Age ≥65	3.53	2.20	4.83	6.46	4.06	8.80	9.53	6.03	12.9
AGB	Age <65	7.09	6.79	7.38	12.79	12.3	13.28	18.55	17.83	19.25
	Age ≥65	4.63	3.86	5.39	8.44	7.08	9.79	12.39	10.42	14.32

*AGB*, adjustable gastric banding; *CI*, confidence interval; *HR*, hazard ratio; *HTE*, heterogeneous treatment effect; *RORI*, operation or intervention; *RYGB*, Roux-en-Y gastric bypass; *SG*, sleeve gastrectomy

### Secondary Long-Term Outcomes

#### Endoscopy

Endoscopy for any reason (diagnostic or therapeutic) was less likely for SG vs. RYGB (AHR, 0.43; 95% CI, 0.38–0.48; *P* < 0.001) and also less likely for AGB vs. RYGB (AHR, 0.36; 95% CI, 0.33–0.39; *P* < .001) (Table 3). The corresponding cumulative rate of endoscopy (95% CI) was highest for RYGB: 3.7% (3.5–3.8%) at 1 year, 6.0% (5.7–6.3%) at 3 years, and 8.3% (7.9–8.7%) at 5 years (Table 4, Fig. 2c).

#### Revision

Revisional procedures appeared to be most common after AGB, followed by SG and then RYGB (AHR of AGB vs. RYGB, 11.3; 95% CI, 10.2–12.5; *P* <0.001; AHR of SG vs. RYGB, 2.9; 95% CI, 2.5–3.3; *P* <0.001) (Table 3). The highest estimated cumulative probability of revision (95% CI) were on AGB patients: 5.5% (5.2–5.8%) at 1 year, 9.3% (8.8–9.8%) at 3 years, and 14.9% (14.1–15.7%) at 5 years.

#### Hospitalization

Hospitalization was less likely after ABG and SG than after RYGB: AGB vs. RYGB, AHR=0.73; 95%CI, 0.71–0.76; *P* <0.001; SG vs. RYGB, AHR=0.79; 95%CI, 0.76–0.83; *P* <0.001 (Table 3). The estimated cumulative incidence rates of hospitalization (95% CI) for RYGB were 14.3% (14.0–14.7%) at 1 year, 30.0% (29.4–30.6%) at 3 years, and 42.3% (41.5–43.0%) at 5 years (Table 4, Fig. 2d).

#### Mortality

For time to all-cause mortality, the AHR was significantly lower after SG than RYGB: AHR, 0.76; 95%CI, 0.64–0.92; *P* =0.004. Compared to RYGB, AGB was associated with lower mortality risk (AHR, 0.49; 95%CI, 0.43–0.56; *P* =0.001) (Table 3). The estimated cumulative risk of all-cause mortality (95% CI) for RYGB was 0.34% (0.30–0.38%) at 1 year, 0.64% (0.57–0.71%) at 3 years, and 0.98% (0.88–1.09%) at 5 years (Table 4).

## Discussion

Our study assessed short- and long-term bariatric surgery risks for adults in a real-world setting utilizing administrative claims data. Patients who underwent RYGB tended to have higher risks for major adverse events as compared to AGB or SG.

Consistent with reported patterns [13, 14, 20], we found a shift away from RYGB toward the use of AGB by the mid-to-late 2000s, while, since 2011, SG rose in preference and became predominant. In the recently published PBS utilizing the same cohort selection criteria, Arterburn et al. reported 5.5% of patients had AGB in their adult sample [21] compared to our finding of 36.0%. This is likely explained by our longer study period and ability to capture bariatric procedures in broader representation of healthcare settings (the EHR data from PBS over-represented academic medical settings). This might also explain the higher percutaneous or operative intervention rate of AGB observed within 30 days after the surgery.

Regarding long-term adverse events, our results show directional similarities with prior studies. Similar to the PBS [22] and other studies [25, 26] that compared the risk of RYGB versus SG, we found RYGB patients were more likely to require operation or intervention and hospitalization than SG patients in the long run. However, different from the PBS, the risks of AGB were smaller in our current study, and we observed a significant association between AGB and lower hospitalization and mortality when comparing to RYGB and SG. This difference may in part reflect a sampling difference in AGB patients. In our study, the AGB patients tended to be followed longer (median 3.3 vs. 1.1 years for SG) and the SG patients had a shorter time to hospitalization (median 0.9 vs. 2.4 years) at follow-up.

Although the estimated cumulative mortality risk was consistently less than 1% up to 5 years after three procedure types, we observed that RYGB patients had a significant higher death rate. Differently, the PBS showed similar mortality rates for SG and RYGB [22]. Of note, the RYGB and SG patients in our study had shorter follow-up time compared to the PBS cohort (median 2.5 vs. 3.4 years for RYGB; median 1.1 vs. 2.2 years for SG). This should be considered in interpreting the results. Additional follow-up time is necessary to examine the long-term mortality differences between bariatric procedures, particularly for SG.

Regarding the risk of revisional procedures, we found AGB patients had higher risk than RYGB and SG patients, consistent with the fact that ABG has been largely abandoned as a bariatric procedure owing to concerns about insufficient weight loss and the need for reoperation due to band failures and slippage. The estimated risk of revision was 14.9% at year 5 for AGB compared to 1.4% for RYGB and 4.0% for SG. This result is consistent with Ibrahim et al., in which 18.5% of Medicare beneficiaries who underwent AGB had reoperations over an average of a 4.5-year follow-up [27]. There was a significantly higher revision risk associated with SG (AHR of 2.88) when compared to RYGB. The corresponding AHR was 1.17 in the PBS but did not reach statistical significance.^24^ Similar to our result, Lewis et al. reported SG patients were more likely to undergo bariatric conversion or revision (AHR, 1.83; 95% CI, 1.19–2.80) [25]. A prior study showed more weight regain after SG at 5 years [21], which could contribute to the higher likelihood of revision associated with SG. Other explanations could be including the planned second-stage conversion (for early-stage SG) and the continuing or worsen of GERD associated with SG [28].

In the heterogeneity test, we found the harm of AGB over RYGB and SG for operation or intervention was greater with females and for those less than 65 years old. This finding suggests that females and younger population do better with RYGB or SG rather than AGB. Our study confirms the heterogeneity findings regarding gender and age in the PBS [22].

When interpreting the results, the different follow-up time of each procedure group is worthy of note. AGB patients had the longest follow-up with a median of 3.3 years, and SG patients had the least with a median of 1.1 years. The difference was tied to the time trends in bariatric procedure evolution. Given the rise of SG towards the end of the study period, our analysis did not have a large number of SG patients followed for 5 years, which could impair the likelihood to distinguish the difference for rare and late-onset outcomes such as revision and mortality. Studies with more longer-term follow-up will be important to examine our findings for SG populations.

Prior studies have presented the comparative effectiveness and safety of bariatric procedures on obese patients and the subset with diabetes [21–25, 29, 30]. Our study adds safety information for bariatric procedures using a large national health plan data. Our results clarify that AGB is associated with high risk of subsequent operation/intervention and revision and becomes less common in practice. Regarding SG and RYGB, our results show that RYGB has a higher risk of subsequent operation or intervention, but SG has a higher revision rate possibly due to more weight regain and reflux. The findings of this study will undoubtedly contribute to the larger understanding needed in making clinical decisions, and also help payers to decide on the best investments in this area.

### Limitations

This observational study relied on secondary data repurposed for research from their original transactional functions. The HCARN data does not provide information on race and ethnicity, nor do they include risk factors such as family history, diet, and exercise regimen—all of which could influence outcomes substantively. The data does not capture the member’s healthcare utilization if they disenroll from the health plans, which leads to loss of follow-up. More directly, anthropometric measures, such as body mass index, were not available in claims data, and comorbidity resolution data was not examined during the follow-up. Additionally, as aforementioned, the SG group had a shorter follow-up time. As a result, these findings must be interpreted with caution. While the study population was reflective of working-age adult patients in commercial health plans, the results may not be generalizable to patients enrolled in different types of health plans, those outside the United States, or those without health insurance.

## Conclusion

This study compared the risks of the three commonly performed bariatric procedures in a large and broadly representative sample. This study showed that AGB patients experienced the most risk of adverse events in general, and RYGB had a higher risk of operation and intervention but sustainable less need for revision comparing to SG after the initial bariatric procedure. Our results extend the PBS results by adding claims data representative of academic and non-academic medical centers, which could provide additional perspectives and guidance, and a better understanding of the range of longer-term outcomes from bariatric procedures.

## Supplementary Information

ESM 1 (DOCX 79 kb)
