# Supplementary material for: Interventions and Operations after Bariatric Surgery in a Health Plan Research Network Cohort from the PCORnet, the National Patient-Centered Clinical Research Network
Source: Obes Surg. 2021 Apr 20;31(8):3531–40. doi: 10.1007/s11695-021-05417-7 (PMC8270856; doi:10.1007/s11695-021-05417-7)
Supplement: Supplementary file 1 — (DOCX 79 kb) [file 11695_2021_5417_MOESM1_ESM.docx]

**Supplemental Digital Content 1,** **Table 1.** ICD-9 and CPT-4 codes used to identify bariatric procedures

| **Code** | **Description** | **Procedure Assignment** | **Code type** |
| --- | --- | --- | --- |
| 43.82 | Laparoscopic vertical (sleeve) gastrectomy | SG | ICD-9 |
| 43.89 | Partial gastrectomy with bypass gastrogastrostomy; Sleeve resection of stomach | SG | ICD-9 |
| 43775 | Laparoscopic sleeve gastrectomy | SG | CPT-4 |
| 44.31 | High gastric bypass; Printen and Mason gastric bypass | RYGB | ICD-9 |
| 44.39 | Other gastroenterostomy; Bypass gastroduodenostomy; gastroenterostomy; gastrogastrostomy; Gastrojejunostomy without gastrectomy NOS | RYGB | ICD-9 |
| 43633 | Gastrectomy, partial, distal; with Roux-en-Y reconstruction | RYGB | CPT-4 |
| 43846 | Gastric restrictive procedure, with gastric bypass, for morbid obesity; with short limb (less than 100 cm) Roux-en-Y gastroenterostomy | RYGB | CPT-4 |
| 43847 | Gastric restrictive procedure, with small intestine reconstruction to limit absorption; with long limb (>150 cm) Roux-en-Y | RYGB | CPT-4 |
| 44.38 | Laparoscopic gastroenterostomy; Bypass: gastroduodenostomy; gastroenterostomy; gastrogastrostomy; Laparoscopic gastrojejunostomy without gastrectomy NEC | RYGB | ICD-9 |
| 43644 | Laparoscopy, surgical, gastric restrictive procedure with gastric bypass and Roux-en-Y gastroenterostomy (roux limb 150 cm or less) | RYGB | CPT-4 |
| 43645 | Laparoscopy, surgical, gastric restrictive with gastric bypass and small intestine reconstruction to limit absorption | RYGB | CPT-4 |
| 43844 | Laparoscopic gastric restrictive procedure with gastric bypass and Roux-en-Y gastroenterostomy | RYGB | CPT-4 |
| S2085 | Lap GASTRIC BYPASS | RYGB | HCPCS |
| 44.95 | Laparoscopic gastric restrictive procedure | AGB | ICD-9 |
|  | Adjustable gastric band and port insertion |  |  |
| 43770 | Laparoscopy, surgical, gastric restrictive procedure: placement of adjustable gastric band | AGB | CPT-4 |
| S2082 | Lap Band | AGB | HCPCS |

AGB=adjustable gastric banding; CPT-4=Current Procedural Terminology, 4th Edition; HCPCS=Healthcare Common Procedure Coding System; ICD-9=International Classification of Diseases, Ninth Revision; NEC=not elsewhere classifiable; NOS=not otherwise specified; RYGB=Roux-en-y gastric bypass procedure; SG=sleeve gastrectomy**Supplemental Digital Content 2, Figure 1.** Patient Flow


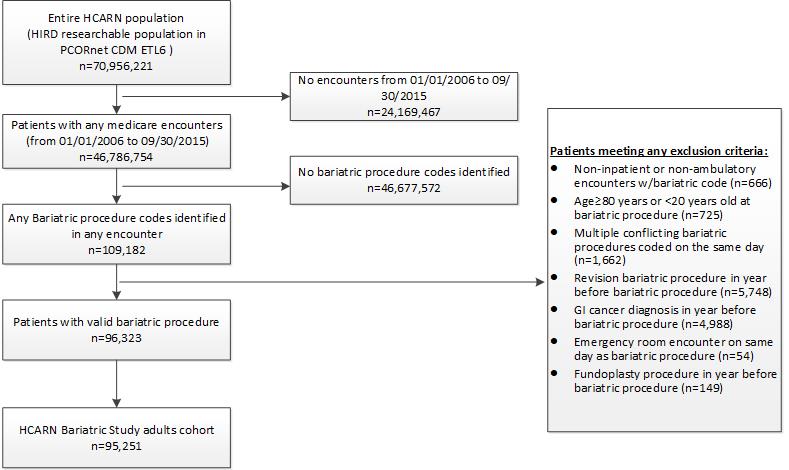


CDM ETL6=common data model data extraction, transformation, and loading, Version 6; GI=gastrointestinal; HCARN= HealthCore-Anthem Research Network; HIRD=HealthCore Integrated Research Database; PCORnet= Patient-Centered Clinical Research Network

**Supplemental Digital Content 3, Table 2.** **Adjusted Odds Ratios and Hazard Ratios for Comparison of Different Events among patients 20-64**

| **Outcome** | **Procedures** | **Adjusted Odds Ratio/hazard ratios** | **Lower 95% CI** | **Upper 95% CI** | **P-value** |
| --- | --- | --- | --- | --- | --- |
| **30-day composite Outcome** | SG vs. RYGB | 0.99 | 0.88 | 1.11 | 0.22 |
|  | AGB vs. RYGB | 0.85 | 0.78 | 0.93 | <0.001 |
|  | AGB vs. SG | 0.86 | 0.76 | 0.97 | <0.001 |
| **Operation or Intervention, excluding endoscopy** | SG vs. RYGB | 0.88 | 0.80 | 0.96 | 0.004 |
|  | AGB vs. RYGB | 2.15 | 2.04 | 2.26 | <0.001 |
|  | AGB vs. SG | 2.46 | 2.25 | 2.68 | <0.001 |
| **Endoscopy** | SG vs. RYGB | 0.43 | 0.38 | 0.48 | <0.001 |
|  | AGB vs. RYGB | 0.36 | 0.33 | 0.39 | <0.001 |
|  | AGB vs. SG | 0.85 | 0.74 | 0.97 | 0.02 |
| **Revision** | SG vs. RYGB | 2.95 | 2.54 | 3.42 | <0.001 |
|  | AGB vs. RYGB | 11.43 | 10.31 | 12.67 | <0.001 |
|  | AGB vs. SG | 3.88 | 3.44 | 4.37 | <0.001 |
| **Hospitalization** | SG vs. RYGB | 0.79 | 0.75 | 0.83 | <0.001 |
|  | AGB vs. RYGB | 0.73 | 0.71 | 0.75 | <0.001 |
|  | AGB vs. SG | 0.92 | 0.88 | 0.97 | 0.002 |
| **Mortality** | SG vs. RYGB | 0.77 | 0.62 | 0.95 | 0.015 |
|  | AGB vs. RYGB | 0.49 | 0.43 | 0.57 | <0.001 |
|  | AGB vs. SG | 0.64 | 0.50 | 0.81 | 0.0003 |
